# Does retrieving a memory insulate it against memory inhibition? A retroactive interference study

**DOI:** 10.1080/09658211.2019.1710216

**Published:** 2020-01-19

**Authors:** Justin C. Hulbert, Michael C. Anderson

**Affiliations:** a Psychology Program, Bard College, Annandale-on-Hudson, NY, USA; b MRC Cognition and Brain Sciences Unit, University of Cambridge, Cambridge, UK

**Keywords:** Retrieval-induced forgetting, inhibitory control, retroactive interference, cue-independent forgetting

## Abstract

Several recent studies suggest that an initial retrieval attempt imbues retrieved memories with special resilience against future interference and other forgetting mechanisms. Here we report two experiments examining whether memories established through initial retrieval remain subject to retrieval-induced forgetting. Using a version of a classical retroactive interference design, we trained participants on a list of A–B pairs via anticipation – constituting a form of retrieval practice. After next training participants on interfering A–C pairs, they performed 0–12 additional A–C anticipation trials. Because these trials required retrieval of A–C pairs, they should function similarly to retrieval practice in paradigms establishing retrieval-induced forgetting. We observed robust evidence that retroactive interference generalises to final memory tests involving novel, independent memory probes. Moreover, in contrast to practising retrieval of A–C items, their extra study failed to induce cue-independent forgetting of the original B items. Together, these findings substantiate the role of retrieval-related inhibitory processes in a traditional retroactive interference design. Importantly, they indicate that an initial retrieval attempt on a competitor does not abolish retrieval-induced forgetting, at least not in the context of this classic design. Although such an attempt may protect against inhibition in some circumstances, the nature of those circumstances remains to be understood.

Retrieving the past modifies memory in at least two ways. On the one hand, behavioural studies have shown that retrieval fosters later retention of the retrieved content and does so more effectively than does simple re-exposure of the same material (Bjork, 1988; Karpicke & Roediger, 2008; Landauer & Bjork, 1978; Rowland, 2014; van den Broek et al., 2016). Neurobiological research has, moreover, found that retrieval elicits special processes that may facilitate the consolidation or reconsolidation of experiences in long-term memory (e.g., Antony, Ferreira, Norman, & Wimber, 2017), reinforcing the view that retrieval renders memories resilient. On the other hand, a complementary body of research establishes that retrieval causes forgetting of competing information that might impede retrieval of a target event. This “darker side” of retrieval, known as retrieval-induced forgetting, is believed to be produced, in part, by inhibitory control processes that isolate the desired trace in memory and that ultimately shape how accessible memories are. Taken together, retrieval’s positive and negative effects suggest that this process shapes the state of memory adaptively, according to patterns in its use (e.g., Bekinschtein, Weisstaub, Gallo, Renner, & Anderson, 2018).

If retrieval evokes special processes that enhance retention, retrieved items must necessarily be resilient in the face of at least some forgetting mechanisms. Consistent with this view, recent findings suggest that retrieval can, indeed, protect retrieved items from retroactive interference arising from novel encoding (Halamish & Bjork, 2011), proactive interference from prior lists (Pastötter, Schicker, Niedernhuber, & Bäuml, 2011), and directed forgetting (Abel & Bäuml, 2016). Critically, several studies have suggested that an initial retrieval attempt may insulate a memory against the inhibitory processes thought to create retrieval-induced forgetting (Kliegl & Bäuml, 2016; Racsmány & Keresztes, 2015). We put this finding to the test using a classical A–B, A–C retroactive interference design in the current experiments. Doing so enabled us to test whether retrieval-related inhibitory processes play a role in retroactive interference (for speculation on this point, see, e.g., Anderson, 2003; Anderson & Neely, 1996; Anderson, Bjork, & Bjork, 1994; Bäuml, 1996). Moreover, the conventional A–B, A–C design, described in greater detail below, involves training participants to form associations between cues and targets via the method of anticipation, which is a form of retrieval practice. If retrieval practice insulates memory items against inhibition, as has been suggested, any forgetting found with these traditional methods may not reflect inhibition, contrary to speculations like those mentioned above. It is therefore important to examine whether retroactive interference exhibits properties suggesting a role of inhibitory processes. In so doing, we provide a further test of whether an initial retrieval insulates items against inhibitory control. We first summarise some foundational findings regarding retrieval-induced forgetting and, separately, the protective effects of retrieval before examining their potential interaction experimentally.

## Retrieval as a cause of forgetting

Retrieval-induced forgetting is often studied using the retrieval-practice paradigm (Anderson et al., 1994). In a typical experiment, participants study category-exemplar pairs (e.g., FOOD-BREAD, DRINKS-SCOTCH, FOOD-CHERRY). Participants are then asked to practise retrieving half of the exemplars from half the studied categories via cues that include a category name and a word stem (FOOD-B__). Finally, after a short delay, participants are asked to recall all the studied exemplars. On this final test, participants recall more of the items that they practised retrieving (FOOD-BREAD) than baseline items from unpracticed categories (SCOTCH). More interestingly, retrieval practice impairs later recall of unpracticed items from practiced categories (CHERRY) relative to baseline items. The finding that selective retrieval impairs the accessibility of related memories, known as retrieval-induced forgetting (Anderson, 2003; Anderson et al., 1994; see Murayama, Miyatsu, Buchli, & Storm, 2014, for a meta-analysis; Storm & Levy, 2012), generalises to a variety of episodically formed associations (Abel & Bäuml, 2012; Anderson & Bell, 2001; Ciranni & Shimamura, 1999; Ortega-Castro & Vadillo, 2012; Spitzer & Bäuml, 2009), and even affects non-studied semantic competitors (e.g., Johnson & Anderson, 2004).

A substantial body of work has sought to understand the mechanisms underlying retrieval-induced forgetting (see Anderson, 2003; Murayama et al., 2014; Storm & Levy, 2012, for reviews and meta-analysis). Several important findings suggest that such retrieval-induced forgetting is driven, in part, by an inhibitory control process engaged during retrieval. For example, when retrieval-practice trials are replaced by opportunities for additional study, forgetting on competing items is usually abolished, a finding known as retrieval specificity (Ciranni & Shimamura, 1999; Hulbert, Shivde, & Anderson, 2012; see Murayama et al., 2014, for a meta-analysis). Retrieval specificity suggests that strengthening practiced items is not itself the primary cause of forgetting, as both retrieval practice and extra-study exposures strengthen the practiced items. Indeed, the degree of strengthening of the practiced items is usually uncorrelated with retrieval-induced forgetting, suggesting that the phenomenon does not reflect simple associative interference (see, e.g., Murayama et al., 2014). Consistent with this possibility, retrieval-induced forgetting has even been found when successful retrieval practice is rendered impossible, suggesting that the mere effort to retrieve a target item induces forgetting of competitors (Storm & Nestojko, 2009; Storm, Bjork, Bjork, & Nestojko, 2006). In contrast, retrieval-induced forgetting has been tied to the tendency for related memories to compete during retrieval practice, a phenomenon known as competition dependence (Anderson, 2003; see Murayama et al., 2014, for a meta-analysis). Not only do brain signals linked to competition predict retrieval-induced forgetting (Kuhl, Dudukovic, Kahn, & Wagner, 2007; Staudigl, Hanslmayr, & Bäuml, 2010), changes in competition levels between the retrieval practice attempts can be used to classify which newly acquired word pairs are recalled after a week-long delay (Rafidi, Hulbert, Brooks, & Norman, 2018).

Another important finding supporting the involvement of inhibition concerns the tendency for retrieval-induced forgetting to generalise to novel test cues, a property known as cue independence (Anderson & Spellman, 1995; Levy & Anderson, 2002; Murayama et al., 2014; Storm & Levy, 2012). For example, practising retrieval of FOOD-BREAD impairs later recall of competitors learned under that same category (CHERRY), regardless of whether they are tested with the original cue under which they were studied (i.e., the “same probe” condition, e.g., FOOD) or with an independent cue (e.g., an alternative category for CHERRY, like RED-C__, designed to be unrelated to the practiced category or exemplars; Anderson & Spellman, 1995; Anderson, Green, & McCulloch, 2000; Hulbert et al., 2012; Murayama et al., 2014; Weller, Anderson, Gómez-Ariza, & Bajo, 2012). The generalisation of retrieval-induced forgetting to independent test cues unrelated to practiced items has been interpreted as an indication that the forgetting reflects inhibition of the competing trace itself and not an interference process specific to the original cue-target association (see Anderson, 2003; Anderson & Spellman, 1995; Weller et al., 2012, for discussions). Interestingly, retrieval-induced forgetting and its key theoretical properties (strength independence, cue independence, retrieval specificity) now also have been demonstrated in another species, with reversible lesions to the rodent prefrontal cortex selectively abolishing retrieval-induced forgetting (Bekinschtein et al., 2018). These findings point to a causal role of prefrontal inhibitory control processes in this forgetting phenomenon. Correspondingly, human neuroimaging evidence not only has found a role of the prefrontal cortex in resolving competition (Kuhl et al., 2007; Wimber, Alink, Charest, Kriegeskorte, & Anderson, 2015), but it also has shown this contribution to decline over successful target retrievals, as competition is resolved and competitors are forgotten (see also Bekinschtein et al., 2018, for evidence of this in rodents).

## Retrieval can promote resilience against retrieval-induced forgetting

Based on the foregoing dynamics, one might predict that making a memory more accessible would render it a stronger competitor during retrieval of related memories and, thus, a more likely target of inhibitory control (Anderson, 2003). If so, retrieval-induced forgetting might increase, consistent with competition dependence (Anderson, 2003). Recently, however, several findings have suggested an intriguing exception to this tendency: Boosting the accessibility of a memory through an initial round of non-selective retrieval practice can eliminate retrieval-induced forgetting. For example, Kliegl and Bäuml (2016) found that subjecting recently encoded items to a single cycle of non-selective retrieval practice rendered those items resilient to later retrieval manipulations that would usually induce forgetting. By modifying the standard retrieval-practice paradigm, they demonstrated that retrieval-induced forgetting of category-cued exemplars fails to emerge when the selective retrieval phase is preceded by an opportunity to recall all of the previously studied items. Swapping out the initial round of non-selective retrieval for a round of extra study exposures, in contrast, offered no such protection from retrieval-induced forgetting. These findings suggest that retrieval had a particularly salutary effect on retention, appearing to insulate items against inhibitory control mechanisms that normally induce forgetting (see also Racsmány & Keresztes, 2015, for a similar finding). If retrieval insulates memory against retrieval-related inhibitory processes, it could provide important insights into both the mechanisms of retrieval-enhancement effects as well as the potential limits of retrieval-induced forgetting.

One account of why retrieval makes retrieved memories resilient is that it may promote greater contextual distinctiveness of their memory traces (Kliegl & Bäuml, 2016). This hypothesis builds on Karpicke, Lehman, and Aue’s (2014) episodic context account of retrieval-based learning. According to Karpicke et al.’s account, intentionally retrieving a past event fundamentally requires the retrieval of contextual information about the original experience that allows a person to isolate that trace in memory. Critically, when this contextual information is retrieved, the item’s contextual representation is updated so that it reflects a combination of the retrieved encoding context and the current temporal context in which retrieval is taking place. This combined contextual representation is stored in memory with the retrieved content, creating a distinctive contextual representation with which a person may later recall the retrieved information once again. Extra study exposures of to-be-learned material, by contrast, are thought less likely to trigger retrieval of the original study context, yielding a less rich contextual representation of the repeated item. This disparity in context retrieval has been proposed to explain why retrieval practice is more effective than extra study exposures in promoting long-term retention.

Building on this hypothesis, Kliegl and Bäuml (2016) proposed that conducting an initial retrieval of studied items ensures that each item is stored with a distinctive context representation. This boost in distinctiveness for each memory item is proposed to reduce the tendency for items sharing a retrieval cue to compete with one another. Given this reduction in competition, inhibitory control processes are no longer necessary to retrieve target items, eliminating retrieval-induced forgetting (Kliegl & Bäuml, 2016). This view is broadly consistent with related proposals arguing that retrieval protects memories against proactive and retroactive interference by promoting greater segregation between competing study lists (Abel & Bäuml, 2014; Bäuml & Kliegl, 2013; Halamish & Bjork, 2011; Szpunar, McDermott, & Roediger, 2008).

Although Kliegl and Bäuml’s (2016) contextual distinctiveness hypothesis fits their findings, several findings in the literature raise concerns that should be addressed. First, the claim that greater distinctiveness of individual items makes them less susceptible to inhibition is inconsistent with certain findings regarding retrieval-induced forgetting. For example, Anderson et al. (2000) found that when to-be-retrieved items and competitors sharing the same cue are rendered more distinctive by a manipulation designed to encourage the incidental encoding of unique properties of the competitors, retrieval-induced forgetting was significantly greater than when participants were encouraged to find similarities between targets and competitors. Anderson et al. (2000) predicted this finding based on the feature-overlap model of retrieval-induced forgetting proposed by Anderson and Spellman (1995). If retrieval practice promotes the distinctiveness of targets and competitors, as Kliegl and Bäuml (2016) claim, these earlier findings suggest that retrieval-induced forgetting should have increased, not decreased.

Second, Kliegl and Bäuml (2016) tested their predictions regarding the insulating properties of retrieval by asking participants to freely recall all studied category exemplars, given the category name as a cue. Although this procedure may facilitate storage of distinctive temporal representations for individual items, as Kliegl and Bäuml maintain, such a procedure is also known to facilitate the development of organised retrieval plans that link items together (e.g., classical work on category clustering by Bousfield, Cohen, & Whitmarsh, 1958; and on subjective organisation by Tulving, 1962; more recently, by Zaromb & Roediger, 2010). This likely confounding of increased item-specific context with list-wide organisation opens Kliegl and Bäuml’s (2016) findings to an alternative account in terms of inter-item integration, a factor that is already known to abolish retrieval-induced forgetting (e.g., Anderson & McCulloch, 1999; Goodmon & Anderson, 2011). If integration underlies Kliegl and Bäuml’s (2016) finding of reduced retrieval-induced forgetting following an initial round of non-selective retrieval, retrieving individual items through cued, rather than free, recall may not provide any special protection against memory inhibition. This possibility led us to ask whether initially retrieving individual competitors, cued one at a time, would insulate items from the inhibition thought to arise during later selective retrieval practice – a design in which inter-item integration was less likely.

## The current study

The current study sought to test whether retrieval inhibits competing memories when those competitors have previously been retrieved in a manner unlikely to promote changes in inter-item integration. To test this possibility, we used a retroactive interference design in which participants learned two lists of word pairs, in which the cues were shared but the responses differed. Specifically, the A–B, A–C paired-associate paradigm measures retroactive interference by requiring participants to learn new associates that share a retrieval cue (A–C pairs, like MOSS-DAMP) with previously acquired associations (A–B pairs, like MOSS-NORTH). Traditionally, pairs in such retroactive interference studies have been trained via the method of anticipation, in which participants learn to anticipate (i.e., retrieve) the response word given the cue, prior to receiving feedback. After anticipation training on the first and then the second list, memory for the original B responses (e.g., NORTH) is then retested with the shared A cues (e.g., MOSS). This procedure typically impairs recall of the original B responses relative to a control in which interpolated learning of A–C items was either absent or involved unrelated (D–E) pairs (see Anderson & Neely, 1996, for a review; Barnes & Underwood, 1959; Briggs, 1954).

We chose to use this retroactive interference procedure to study the insulating effects of retrieval for three reasons. First, because this procedure incorporates retrieval of learned material in the context of individual pairs, it enables us to separate any impact that retrieval has on item-specific context encoding from changes in list-wide organisation. Second, it positions us to address the potential protective effects of retrieval across a range of burgeoning literatures centred on interference phenomena, including memory reactivation (for reviews, see Scully, Napper, & Hupbach, 2017; Xue, 2018), sleep consolidation (e.g., Ellenbogen, Hulbert, Stickgold, Dinges, & Thompson-Schill, 2006; Mednick, Cai, Shuman, Anagnostaras, & Wixted, 2011), and reconsolidation processes (e.g., Lee, Nader, & Schiller, 2017). Finally, because the case for a role of inhibition in retroactive interference has not been empirically developed, our choice of paradigm enabled us to examine whether this phenomenon exhibits the core characteristics of retrieval-induced forgetting taken to reflect inhibition. We, therefore, adapted the traditional retroactive interference design with anticipation training to test whether critical properties such as cue independence and retrieval specificity arise for items that had been trained initially through retrieval-based anticipation learning.

In our procedure, participants first studied A–B pairs, receiving at least one anticipation trial per item prior to studying a list of A–C pairs. Participants then performed anticipation trials on the second-list pairs, attempting to recall each C word aloud, given the A cue either 0 (baseline), 1, 6, or 12 times. In Experiment 2, half of the participants performed anticipation trials on A–C pairs; the remaining half were instead re-exposed to the intact A–C pairs for the same number of times, without the need to retrieve the A–C item. In both experiments, we then administered two final recall tests for B items: the conventional same-probe (SP) test, in which participants received the A cue and had to recall B,^1^ and the independent-probe (IP) test, in which they instead received cues composed of an extra-list associate of B, along with a word stem. Retroactive interference could be said to have arisen if we observed reliably worse first-list recall performance for items whose A–C counterparts received additional anticipation learning (the 1, 6, and 12 repetitions conditions) compared to items in the baseline condition (the 0-repetition condition), with impairment growing as the number of anticipation trials on A–C items increases. If inhibitory processes contribute to retroactive interference, some component of the impairment should generalise to the IP test, demonstrating a contribution of cue-independent forgetting (Anderson, 2003; Anderson & Spellman, 1995). A finding of cue-independent retroactive interference in the context of this paradigm would suggest that an initial retrieval attempt (in the form of anticipation training) on A–B items does not fully insulate those competitors from inhibition. Alternatively, a finding of cue-specific retroactive interference would support the view that inhibitory processes are rendered unnecessary by the storage of distinctive context information generated through a retrieval-based training (Kliegl & Bäuml, 2016). Whereas a failure to find retroactive interference on either final test would be consistent with the predication that retrieval has the power to insulate against interference more generally (Halamish & Bjork, 2011).

## Experiment 1

### Method

#### Participants

Thirty-two University of Oregon undergraduates participated to fulfil a course requirement. Three participants not included in the above count were tested but excluded from analyses and replaced in accordance with posted eligibility criteria and laboratory standards due to (one each): lack of sleep, being a non-native English speaker, or failure to comply with instructions based on a post-experiment questionnaire. The sample size was determined by counterbalancing and decades of experience using related retrieval-induced forgetting paradigms (e.g., Anderson et al., 1994). To retrospectively evaluate our pre-determined stopping rule, we subsequently conducted a power analysis using G*Power3.1 (Faul, Erdfelder, Lang, & Buchner, 2007) indicated that a total sample of 30 participants would be sufficient to have 95% power (using an alpha of .05) for detecting a within-participants cue-independent retrieval-induced forgetting effect commensurate with the effect size (Cohen’s *f* = 0.406) reported by Hulbert et al. (2012). For the purposes of counterbalancing, we established a target sample of 32 participants.

#### Design

We manipulated both retrieval practice (0, 1, 6, or 12 repetitions of the A–C anticipation task) and test type (same-probe, SP, and independent-probe, IP, tests) as within-participants factors. The percentage of first-list responses correctly recalled was measured for each of the two test types.

#### Materials

##### First-list Pairs

We constructed two lists, each with 24 critical noun pairs composed of a cue and a response (e.g., MOSS-NORTH) and 10 filler noun pairs. A single list of critical pairs constituted the first-list (A–B) learning material for all participants. We selected cues and responses for each pair to meet the following conditions: (a) each had no more than nine letters or three syllables; (b) first-list response words represented a member of a unique category (e.g., the category DIRECTION for the response NORTH); (c) cues lacked strong, pre-existing associations (Nelson, McEvoy, & Schreiber, 2004) to either their corresponding responses or to any other item in the word pool, save for their corresponding IP category cues.

##### Second-list Pairs

The second list (A–C) included pairs composed of first-list Cues and novel Response words (e.g., MOSS-DAMP). We chose these novel responses (C items) to be as relatable to the shared cue (A) as were their first-list counterparts (e.g., B items), while ensuring that there was no measured relationship between the two responses themselves, according to association norms (Nelson et al., 2004). As with first-list responses, we minimised links between these novel response words and all other items in the stimulus set. Additionally, for each of the 24 critical cue words, the two associated responses began with different letters.

#### Procedure

##### First learning list

We instructed participants to form an association between paired words presented centrally on a computer monitor for 6 s, such that they would later be able to recall the right-hand word, given the left-hand word. Two filler pairs bounded each end of the stimulus list to control for primacy and recency effects. To ensure that the critical A–B pairs comprising the competitors for our four repetition conditions during the A–C list (0, 1, 6, 12) did not differ in their learning treatment, we randomly divided them into quarters and block-randomised their appearance in the learning sequence; this ensured that equal number of items from each of the four repetition conditions occurred in every learning block, matching the serial position of pairs across these conditions.

**Figure 1.**
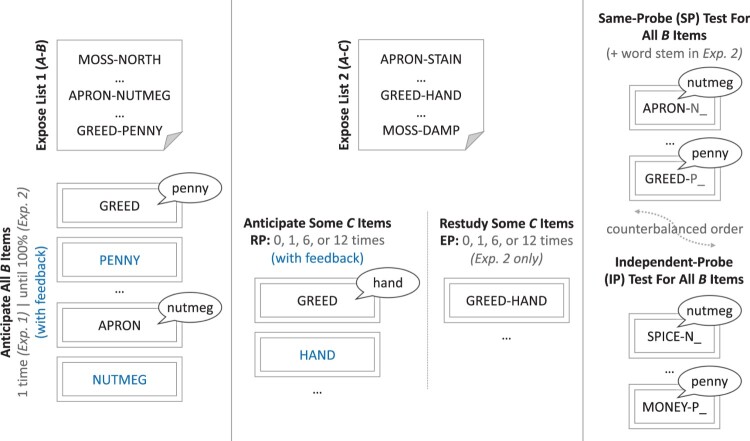
Overview of the basic procedure for Experiments (Exp.) 1 and 2. *Left panel:* In the first phase of the procedure, participants were exposed to an initial list of word pairs (designated A–B) before they were prompted to audibly retrieve the right-hand associates given a randomised sequence of left-hand cues in anticipation of the correct response presented in blue. Rather than a single round of anticipation, participants in Exp. 2 continued this procedure until each response was correctly retrieved once. *Middle panel:* In the second phase, participants were exposed to a new list of pairings, in which the cues (A items) were shared with List 1 but the associates were novel (C items). Participants were then prompted to selectively engage in retrieval practice (RP) of the C items from a subset of the newly exposed pairings 1, 6 or 12 times each, with the remaining critical items held out to form a baseline (the 0 condition). Half of the participants in Exp. 2 were instead given extra practice (EP group) in the form of an equivalent number of passive restudy opportunities with the intact pairings. *Right panel:* Finally, participants were given two recall tests for the original B items, with the test order counterbalanced across participants. One test utilised the same probes (SP) as were learned in the first list to cue recall; the other test instead used independent probes (IP), which were composed of extra-list semantic category cues, together with the first letter of the associate. Word stems were similarly provided in the SP test for Exp. 2.

Next, we asked participants to say aloud the learned associate in response to each centrally presented cue word within a 3 s window. We presented these test cues in a re-randomised blocked presentation order, similar to that used at encoding. Following each trial, we presented the correct answer for each cue, as is customarily done with the method of anticipation. A 300-ms inter-trial interval ensued, during which we displayed a fixation cross. We tested each A–B pair with this anticipation method once during this phase. See Figure 1 for an overview of this phase, along with the rest of the procedure.

##### Second learning list

We presented the second set of pairs in a similar manner, with the additional constraint that no A–C pair be presented in the same serial position as its corresponding A–B pair (McGeoch & McKinney, 1937). We warned participants that the second list would include cues from the first list and that they should avoid providing first-list responses during the second-list anticipation/retrieval practice phase (described below).

##### Retrieval practice phase

Retrieval practice (i.e., A–C anticipation) trials commenced in a manner similar to that used during first-list learning, though we tested some items multiple times, and others, not at all – a fact of which we made participants aware. We divided the 24 critical, second-list pairs into four subsets of six, assigning each to one level of retrieval practice (0, 1, 6 or 12). We counterbalanced the assignment of item sets to retrieval practice conditions across participants, ensuring that every pair participated equally often in each condition. Retrieval practice occurred in a block-randomised fashion, ensuring equal distribution of items from the 1, 6, and 12 conditions across serial positions, with the first and last two trials involving fillers.

##### Final test phase

We administered two final tests in a counterbalanced order, across participants. On each test, we asked participants to verbally recall the first-list response word for each centrally located probe cue. In the SP test, participants received trials presenting the original cue word for up to 4 s. As in the earlier phases, we matched the average serial position on this test for items from each of the four retrieval practice conditions through blocked randomisation. To allow participants to adjust to the task, we tested them on filler items in the first four positions. The IP test’s instructions and presentation order paralleled those used for the SP test, except that each test trial instead presented an extra-list semantic category together with a single-letter word stem of a given first-list response word (e.g., DIRECTION – N__ for NORTH), rather than presenting the originally studied cue word as a probe.

### Results

We submitted recall accuracy on the final tests to a mixed factorial ANOVA, with test order (SP- or IP-first) and item counterbalancing as between-participants factors, and type of test (SP or IP) and level of retrieval practice (0, 1, 6, or 12 repetitions) as within-participants factors. There were no significant interactions of test order or counterbalancing with any effects of interest. We used a Greenhouse-Geisser correction for apparent violations of sphericity. We went on to covary out mean recall during first-list anticipation, though all means are reported in their raw form. The improved power provided by this ANCOVA yielded results consistent with the standard ANOVA, often with increased reliability.

#### First-list anticipation

The four stimulus subgroups showed comparable first-list anticipation performance, *F*s < 1. As such, differences in first-list recall following retrieval of interfering materials were unlikely to be due to pre-existing training imbalances.

#### Retrieval practice success

After studying the second list, participants practised retrieving the second-list response words up to 12 times on an anticipation test that was otherwise identical to that used during first-list learning. Recall accuracy during retrieval practice (with parenthetical standard deviations) increased with the number of retrieval practice attempts: 1 attempt = 41.1% (23.6), 6 attempts = 83.9% (13.2), and 12 attempts = 89.7% (9.29); *F*(1.40,33.51) = 157.19, MSE = .02, *p* < .001, $\eta_{p}^{2}$_ _= .87, 90% CI [.78, .90].

#### Final test performance

To identify retroactive interference, we compared recall of first-list responses as a function of whether their corresponding second-list responses received 0, 1, 6, or 12 retrieval practice trials. We found that increasing numbers of retrieval practice attempts on A–C pairs impaired overall recall of first-list items, ANOVA: *F*(3,72) = 6.70, MSE = .04, *p* < .001, $\eta_{p}^{2}$_ _= .22, 90% CI [.07, .32]; ANCOVA: *F*(3,71) = 9.46, MSE = .03, *p* < .001, $\eta_{p}^{2}$_ _= .29. Overall, participants recalled more first-list items on the IP test (M = 70.3%; SD = 18.7) than on the SP test (M = 47.7%; SD = 19.4), ANOVA: *F*(1,24) = 53.80, MSE = .06, *p* < .001, $\eta_{p}^{2}$_ _= .69, 90% CI [.48, .78]. Test type did not interact, however, with any factors of theoretical interest, *F*s < 1. Nevertheless, we examined recall performance separately for the SP and IP tests and characterise the associated rates of forgetting on the two measures.

**Figure 2.**
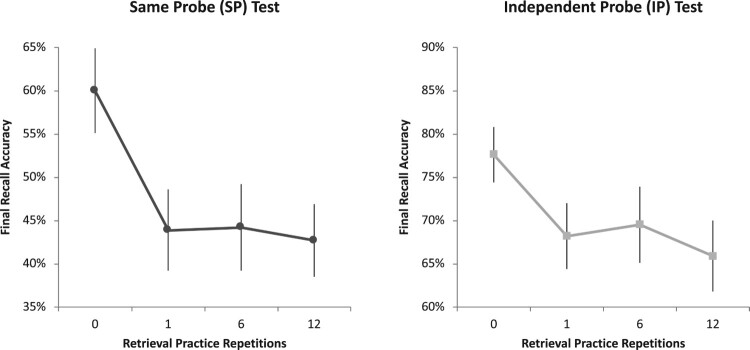
Final recall accuracy of first-list items in Experiment 1 as a function of the number of times the second-list counterparts had undergone retrieval practice. Retrieval practice yielded reliable forgetting on both the same-probe (SP; *left panel*) and independent-probe (IP; *right panel*) tests, indicative of cue-independent forgetting. For visualisation purposes, the *y*-axes were re-windowed across the two panels using a constant 30% range to highlight the effect of retrieval practice on recall, rather than the main effect of test type. Error bars represent standard error of the mean.

As can be seen from the left-hand panel of Figure 2, first-list recall on the SP test exhibited a significant negative linear trend as the level of retrieval practice on list-two responses was increased, *F*(1,24) = 9.70, MSE = .04, *p* = .005, $\eta_{p}^{2}$_ _= .29, 90% CI [.06, .48].^2^ Thus, manipulating the degree of A–C retrieval generated retroactive interference under typical testing conditions. We observed a similar result in the corresponding ANCOVA, linear component: *F*(1,23) = 16.54, MSE = .03, *p* < .001, $\eta_{p}^{2}$_ _= .42. Critically, on the IP test, first-list recall also exhibited a significant negative linear trend as the level of retrieval practice on list-two responses was increased, ANOVA: *F*(1,24) = 10.99, MSE = .02, *p* = .003, $\eta_{p}^{2}$_ _= .31, 90% CI [.08, .50]; ANCOVA: *F*(1,23) = 10.41, MSE = .02, *p* = .004, $\eta_{p}^{2}$_ _= .31, even though we tested memory with a novel cue, unrelated to the practiced competitor (see the right-hand panel of Figure 2).

Despite the overall increase in retroactive interference with increasing levels of retrieval practice, we failed to detect further increases in retroactive interference between the 6 and 12 repetitions conditions, relative to the single-repetition condition on either test type, *F*s < 1. Nevertheless, we observed robust retroactive interference separately at every level of repetition when compared to the baseline condition, a finding remarkably similar on both test types, *p* < .005 and *p* < .02 for all retrieval levels on the SP and IP tests, respectively.

### Discussion

Two main findings emerged from Experiment 1. First, Experiment 1 demonstrated retroactive interference using a within-subjects A–B, A–C interference paradigm even though A–B pairs were trained using anticipation learning. The retrieval attempts made during anticipation were apparently not sufficient to protect the material from retroactive interference. When later provided with the studied cue (A) and asked to recall the first-list response (B), participants tended to forget items whose second-list counterparts (C) received one or more retrieval practices. Secondly, and crucially, we observed similar forgetting on the independent-probe test, and the magnitude of this forgetting effect did not interact with test type. Because the IP test employs a cue designed to be unrelated to the original study cue, the potential contribution for cue-dependent interference mechanisms such as associative blocking is greatly reduced. As such, this cue-independent forgetting is consistent with the hypothesised contribution of inhibition to retroactive interference. Thus, although interference of this sort was often explained historically by the tendency for the studied cue to persistently elicit the stronger, second-list associate (McGeoch, 1942), the current findings suggest that such a mechanism is not sufficient to account for the phenomenon.

Although the current findings point to a contribution of inhibition to retroactive interference, performance on the SP test could reflect a combination of inhibition, blocking, and associative unlearning – a possibility consistent with the numerical (though not reliable, *F* < 1) tendency for greater retroactive interference on the SP test (M = 17%) than on the IP test (M = 12%). Another account of this numerical difference in forgetting across tests relates to the degree to which the two types of test probe disambiguate possible responses: The IP test included a distinctive word stem as an additional cue, whereas the SP test – like many older tests of retroactive interference – did not. Indeed, previously we have used such distinctive item-specific stem cues expressly because their highly constraining nature focuses retrieval effectively; they have been used to reduce covert cueing strategies (Anderson et al., 2000), and output interference in studies of retrieval-induced (see Anderson, 2003; Murayama et al., 2014; Storm & Levy, 2012, for reviews). The lack of an item-specific word stem on our SP test could have made it easier for participants to mistakenly provide a second-list item during final retention test of the first list. Thus, some of the forgetting may represent source (list) confusion. In Experiment 2, we therefore, considered whether providing an item-specific stem might reduce retroactive interference on the SP test so that it was similar in size to that observed on the IP test.

#### Determining retrieval specificity of retroactive interference

Although Experiment 1 found evidence for cue-independent forgetting in retroactive interference, it did so using the traditional method of anticipation, which inherently confounds retrieval practice and interpolated learning, as we have noted previously (Anderson, 2003; Anderson et al., 1994). Separating these factors is theoretically important to determine whether the cue-independent forgetting arises specifically from retrieval-related inhibitory processes or from the strengthening of the association between A and C, irrespective of retrieval. If inhibition underlies cue-independent retroactive interference, forgetting should selectively arise when retrieval practice is performed, and not simply from strengthening due to the feedback component of the method of anticipation. If cue-independent retroactive interference arises from a strength-based learning process, however, forgetting should be observed even under re-exposure conditions not involving retrieval practice.

*Retrieval-specificity* has regularly been found to be a reliable property of retrieval-induced forgetting, especially when the comparison involved extra presentations (Murayama et al., 2014). Electrophysiological and neuroimaging studies also support the notion that retrieval-induced forgetting arises from neural processes distinct from those involved in strengthening practised items (Johansson, Aslan, Bäuml, Gabel, & Mecklinger, 2007; Kuhl et al., 2007; Staudigl et al., 2010; Wimber, Rutschmann, Greenlee, & Bäuml, 2009). Some initial attempts have been made to separate out retrieval practice and degree of interpolated study (Bäuml, 1996; Delprato, 2005), but we are unaware of any demonstration of the retrieval-specific nature of retroactive interference when it is measured using an independent probe. Experiment 2 therefore not only sought to replicate Experiment 1, but also to examine whether evidence for cue-independent forgetting also occurs when retrieval practice is replaced with additional study exposures that don’t require retrieval of A–C pairs. Demonstrating that cue-independent forgetting occurs for retrieval practice, but not for extra study exposures, would provide strong converging evidence for an inhibitory contribution by retrieval, further substantiating the interpretation of Experiment 1 that an initial retrieval attempt does not fully protect against inhibition.

## Experiment 2

### Method

#### Participants

Sixty-four undergraduates (32 in each practice group) from the University of St. Andrews participated to fulfil a course requirement or for payment. We also tested four participants not included in the above counts (two from each the Retrieval Practice (RP) and Extra Practice (EP) groups); experimenter or computer error forced their replacement prior to any analysis. We excluded a single participant (from the EP group) from analyses after data collection had ended due to a failure to comply with instructions, as indicated by self-report on the post-experimental questionnaire. As before, the sample size was determined by counterbalancing and previous experience using related paradigms, including those yielding similar interaction effects (e.g., Anderson et al., 1994). To retrospectively evaluate our pre-determined stopping rule, again we subsequently conducted a power analysis using G*Power3.1 (Faul et al., 2007) indicated that a total sample of 48 participants (24 in each group) would be sufficient to have 95% power (using an alpha of .05) for detecting an interaction of practice type by practice level commensurate with the effect size (Cohen’s *f* = 0.299) reported by Hulbert et al.’s (2012) study of cue-independent retrieval-induced forgetting.

#### Design

As with the previous experiment, we manipulated practice level (0, 1, 6, or 12 A–C repetitions) and test type (SP and IP tests) within-participants. Additionally, we manipulated practice type between participants (retrieval practice vs. extra study practice, hereinafter, RP vs. EP).

#### Materials

We used the same materials as in Experiment 1.

#### Procedure

Experiment 2 proceeded as did Experiment 1, with the following exceptions:

##### Drop-off phase

After the initial presentation of the first learning list, we asked participants to verbally recall each associated B-response given a centrally presented, white A-cue, within a 6 s window. Regardless of the response, we presented the correct answer in blue for 2 s, following a 200-ms inter-stimulus interval. An 800-ms inter-trial interval and 200-ms fixation period preceded every trial. Cues for which a correct response had been generated would drop off of the test list. We then re-randomised and re-tested the remaining items later in order to ensure that every item had been recalled successfully exactly once by the end of the phase.

##### Practice phase

Practice instructions varied by group. Members of RP group received the exact same instructions given in Experiment 1. Members of the EP condition, on the other hand, were informed just prior to the practice phase that they would be given the chance to review intact word pairs by silently reading to themselves whilst avoiding “quizzing oneself” (i.e., covert retrieval practice). We assessed adherence to these rules with a post-experimental questionnaire.

##### Final test phase

As in Experiment 1, we administered the SP and IP tests in a counterbalanced order, across participants. The IP test was exactly the same as before; the SP test, however, incorporated an additional single-letter word stem. We did this to better match the different test types and to reduce the chance that the studied cue would elicit immediately the stronger, second-list response, blocking the first-list response. By design, the IP should bypass the issue of source confusion.

### Results

We submitted recall accuracy on the final tests to a mixed factorial ANOVA, with practice type (EP or RP), test order (SP- or IP-first), and item counterbalancing as between-participants factors. We manipulated type of test (SP or IP) and the level of retrieval practice (0, 1, 6, or 12 repetitions) as within-participants factors. We used a Greenhouse-Geisser correction for apparent violations of sphericity. Because we trained participants to a 100% correct criterion using a drop-off training procedure, we equated initial learning across groups for first-list items prior to the practice phase. Thus, it was not possible to covary out first-list learning as in Experiment 1.

#### Retrieval practice success

Due to experimenter error, we did not record retrieval practice success for one participant in the RP condition. Otherwise, mean retrieval accuracy (with parenthetical standard deviations) for this group increased with the number of retrieval practice attempts as in Experiment 1: 1 attempt = 50.2% (24.2), 6 attempts = 87.3% (8.7), and 12 attempts = 93.2% (5.0); *F*(1.20,27.59) = 102.06, MSE = .03, *p* < .001, $\eta_{p}^{2}$_ _= .82, 90% CI [.69, .87]. Given the nature of the practice performed, there are no corresponding data in the EP condition to report.

#### Final test performance

We observed a robust main effect of the number of practice attempts (regardless of type) on final recall, *F*(3,141) = 9.78, MSE = .02, *p* < .001, $\eta_{p}^{2}$_ _= .17, 90% CI [.08, .25]. Overall, the effect of practice attempts did not interact reliably with the type of practice (RP or EP) performed on the interpolated list, *F*(3,141) = 1.72, MSE = .02, *p* = 0.167, $\eta_{p}^{2}$_ _= .04, 90% CI [< .01, .08], indicating that when we collapsed over the two test types, performance declined with both types of practice. Neither was the three-way interaction (with test type) significant, *F*(3,141) = 1.46, MSE = .02, *p* = .229, $\eta_{p}^{2}$_ _= .03, 90% CI [< .01, .07]. Nevertheless, to test our *a priori* predictions and facilitate the comparison to the results from Experiment 1, we next moved to interrogate the pattern of final test recall for the retrieval practice and extra study exposures conditions separately.

##### Retrieval practice (RP) group

Increasing the number of retrieval attempts on A–C pairs impaired recall of first-list items when performance was collapsed over test type, *F*(3,141) = 5.37, MSE = .02, *p* = .002, $\eta_{p}^{2}$_ _= .10, 90% CI [.03, .17], replicating Experiment 1. The added specificity offered by including a letter stem on the SP test in this experiment reduced, but did not eliminate the observed main effect recall advantage for the IP test (M = 91.0%; SD = 7.0) over the SP test (M = 76.8%; SD = 12.6), *F*(1,47) = 61.83, MSE = .02, *p* < .001, $\eta_{p}^{2}$_ _= .57, 90% CI [.40, .67] (main effect of test type in Experiment 2). As before, the detrimental effects of retrieval practice on the recall of first-list responses did not interact with test type, *F*(3,141) = 1.37, MSE = .02, *p* = .256, $\eta_{p}^{2}$_ _= .03, 90% CI [.01, .07]. Nevertheless, because performance on these tests was of *a priori* interest, we examined recall performance separately for the SP and IP tests.

**Figure 3.**
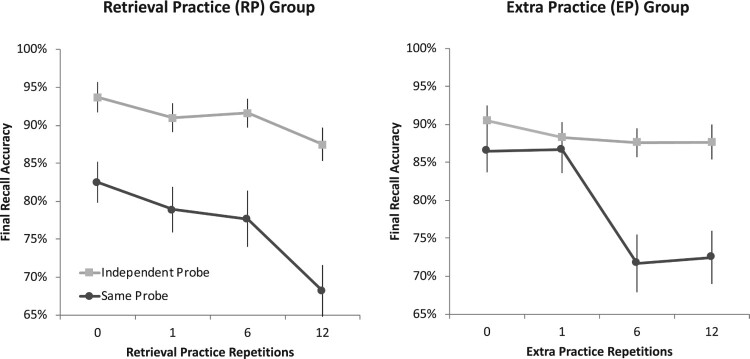
Final recall accuracy of first-list items in Experiment 2 as a function of the number of times the second-list counterparts had undergone retrieval practice (RP group; *left panel*) or extra practice (EP group; *right panel*). Estimated marginal means are accompanied by error bars representing standard error of the mean. As in Experiment 1, retrieval practice yielded evidence of cue-independent forgetting on the independent-probe (IP) test. In contrast to retrieval practice, extra practice led only to reliable forgetting on the same-probe (SP) test.

As the left side of Figure 3 illustrates, first-list recall on the SP test declined linearly with increasing numbers of retrieval practice trials on list-two responses, *F*(1,47) = 9.56, MSE = .03, *p* = .003, $\eta_{p}^{2}$_ _= .17, 90% CI [.04, .32]. Thus, manipulating retrieval of interfering materials generated retroactive interference under typical testing conditions. Critically, first-list IP recall similarly exhibited a linear decline as the level of retrieval practice on list-two responses was increased, *F*(1,47) = 4.46, MSE = .01, *p* = .040, $\eta_{p}^{2}$_ _= .09, 90% CI [< .01, .23], replicating evidence for cue-independent retroactive interference observed in Experiment 1.

##### Extra practice (EP) group

Similar to what we found with the retrieval practice condition, extra presentations of A–C items impaired memory for first-list responses when performance was examined collapsed over test type, *F*(3,141) = 6.00, MSE = .02, *p* = .001, $\eta_{p}^{2}$_ _= .11, 90% CI [.03, .18]. Moreover, overall recall accuracy once again was significantly higher on the IP test (M = 88.7%; SD = 8.9) than it was on the SP test (M = 79.1%; SD = 9.4), *F*(1,47) = 27.88, MSE = .04, *p* < .001, $\eta_{p}^{2}$_ _= .37, 90% CI [.19, .51]. Despite these similarities to retrieval practice, the right side of Figure 3 nevertheless reveals that the impact of extra presentations of A–C items on final recall of A–B items varied significantly with test type, *F*(3,141) = 5.80, MSE = .02, *p* = .001, $\eta_{p}^{2}$_ _= .11, 90% CI [.03, .18]. We explored this interaction further by examining recall performance separately for the SP and IP tests.

On the SP test, first-list recall also exhibited a significant negative linear trend as the level of extra practice on list-two responses was increased, *F*(1,47) = 15.36, MSE = .03, *p* < .001, $\eta_{p}^{2}$_ _= .25, 90% CI [.08, .40]. Critically, however, first-list IP recall did not decline in a similar manner, *F*(1,47) = 0.86, MSE = .01, *p* = .359, $\eta_{p}^{2}$_ _= .02. Thus, the retroactive interference owing to extra presentations appeared to be cue-dependent, unlike that produced by retrieval practice. This finding is consistent with the possibility that cue-independent retroactive interference is specifically caused by retrieval.

### Discussion

The retrieval practice undertaken by participants in Experiment 2 replicated evidence for cue-independent retroactive interference observed in Experiment 1, even after introducing additional controls to ensure that participants learned first-list associations to a 100% criterion and were provided word stems on both final tests to minimise source-memory confusion. The inclusion of word stems on the SP test trials did not abolish the difference in the size of the forgetting effect across SP and IP tests. This finding suggests that a distinct (and likely non-inhibitory) source of retroactive interference may enlarge the forgetting effect on the SP test, and that this effect is unlikely to be due to simple source confusion. If participants truly had been able to recall first and second-list responses and were confused about which answer to provide, the letter stem would have clarified which response we were seeking.

While a single round of retrieval has been sufficient to shield against retrieval-induced forgetting in some previous studies (e.g., Kliegl & Bäuml, 2016; Racsmány & Keresztes, 2015), investigations of the beneficial effect of testing often involve repeated initial retrieval attempts (e.g., Halamish & Bjork, 2011; Potts & Shanks, 2012). Potts and Shanks (2012), for instance, required participants correctly retrieve each response twice (with feedback) in a blocked learning paradigm, which was followed by two additional rounds of retrieval attempts. Certain published evidence suggests that when the original materials are not over-trained in this manner, initial retrieval fails to protect memories from retroactive interference (Hupbach, 2015). To these findings we add our current observation that, despite using anticipation learning to drill participants on first-list responses to perfect performance, cue-independent retroactive interference was found in the RP condition. Results such as these help to establish that initial retrieval does not necessarily insulate items against inhibition fully, at least not in the context of this classical retroactive interference design.

Experiment 2 also incorporated a between-participant manipulation designed to test whether cue-independent retroactive interference derives specifically from retrieval processes or is instead a consequence of any form of memory strengthening on competing A–C associations. We found that extra presentations, like retrieval practice, impaired later recall of related first-list words when tested with the SP test. A qualitatively different pattern was observed on the IP test, however. Specifically, using independent probes on the final test eliminated any reliable sign of a practice-based decrement in the extra presentations condition. Though somewhat tempered by the lack of a reliable interaction effect, this general pattern suggests that cue-independent forgetting in the A–B, A–C retroactive interference paradigm may be specific to retrieval, as it is in the retrieval-induced forgetting paradigm (Hulbert et al., 2012). In contrast, when other forms of interpolation are used, such as extra study, retroactive interference may primarily be a cue-dependent forgetting phenomenon, potentially reflecting associative interference. Future work taking advantage of greater statistical power promises to speak further to this particular claim. Notably, the occurrence of this interference effect indicates that an initial retrieval attempt does not fully insulate against non-inhibitory associative interference processes, in contrast to prior claims (e.g., Halamish & Bjork, 2011).

## General discussion

Four main findings emerge from the current experiments. First, both Experiments 1 and 2 show that intentionally retrieving individual memory items via recently encoded cues does not wholly insulate the retrieved items from the retroactive interference that typically follows learning to anticipate a set of related memories. Using a classical A–B, A–C retroactive interference paradigm with arbitrary word pairs, we found that first-list (A–B) items grew increasingly less well recalled as their competing A–C items on the second list were repeatedly trained using the method of anticipation. This impairment arose despite the fact that all of the A–B associations on the first list were not only studied but were also trained via the method of anticipation – a method that prominently involves an attempt to retrieve each cued item. Moreover, differing levels of anticipation training on A–B associations appeared not to make a measurable difference to the occurrence of retroactive interference: Whereas first-list items all underwent a single anticipation trial in Experiment 1, pairs were trained to a 100% correct criterion in Experiment 2. Notably, the observation of retroactive interference with this design is not, in itself, new. The overwhelming majority of interference studies from the classical interference era (circa 1900–1970) trained items using the method of anticipation, as we have done here. Thus, a large body of work already suggests that retrieving individual items does not completely protect against later interference.

Second, the results from both Experiments 1 and 2 together represent what is, to our knowledge, the first demonstration that retroactive interference in the A–B, A–C design reflects forgetting that generalises to novel retrieval cues – at least when the A–C list is trained via the retrieval-based method of anticipation. Regardless of whether we tested the target items from the A–B pairs with the originally trained cues (i.e., the A cues) or with a novel, extralist semantic cues that were unrelated to the original studied cues (i.e., independent probes), forgetting increased linearly with the number of anticipation trials on A–C items, with no evidence of an interaction of this forgetting pattern with test type. Cue-independent forgetting is a hallmark of retrieval-induced forgetting and has been taken to reflect the impact of inhibition on competing memories (Anderson, 2003; Anderson & Spellman, 1995; Murayama et al., 2014; Storm & Levy, 2012). These findings suggest that training A–C pairs with the method of anticipation, as was done ubiquitously in the interference era, generates forgetting with functional properties similar to retrieval-induced forgetting and consistent with inhibition. Although the possibility that retrieval-induced forgetting may underlie effects in this older literature has been discussed (e.g., Anderson, 2003; Anderson et al., 1994; Anderson & Bjork, 1994; Anderson & Spellman, 1995; Bäuml, 1996), this extrapolation of cue independence has not been explicitly tested. The success of this extrapolation was certainly not guaranteed, especially given the differences in materials (unrelated associates) and temporal structure (two distinct list contexts) that might have altered the mechanisms involved.

Third, the findings from Experiment 2 indicate that cue-independent forgetting in the retroactive interference design likely arises from the retrieval requirement of the method of anticipation. Fundamentally, this traditional method requires that participants anticipate the correct response to a stimulus by retrieving it and saying it aloud prior to receiving feedback on the correct answer. This anticipation process is analogous to a retrieval practice trial in the basic paradigm typically used to study retrieval-induced forgetting, except that in anticipation (a) corrective feedback is always given after each anticipation, (b) no word stem is provided for the correct response, forcing participants to rely on the shared stimulus (e.g., the A cue), and (c) accuracy, at least during A–C training, is determined by whether the response comes from the second list, forcing participants to discriminate the correct items with the temporal context of that list. Because this method confounds episodic retrieval and additional encoding of A–C items during feedback, we sought to determine which factor might drive cue-independent forgetting. We discovered that when participants did not need to retrieve the second-list response during interpolated learning and could simply strengthen the A–C association through extra study exposures, cue-independent forgetting on our independent-probe test disappeared. These findings suggest that the retrieval component of anticipation attempts was critical to inducing cue-independent forgetting, consistent with the view that inhibitory processes are engaged to resolve retrieval competition (Anderson, 2003; Anderson & Spellman, 1995). These findings parallel results from the related retrieval-practice paradigm, which demonstrate that cue-independent forgetting only arises with retrieval practice, and not extra study practice (Hulbert et al., 2012).

Finally, Experiment 2 provides suggestive evidence that extra study exposures induce a qualitatively different pattern of forgetting than does retrieval practice. Whereas retrieval practice impaired performance on both final tests, extra study exposures only impaired memory on the same-probe test of A–B pairs, revealing that forgetting induced by this method of interpolation is cue-dependent. Extra study exposures induced forgetting on the same-probe test despite the addition of a distinctive letter stem for the first-list (B) item on that test. To the extent that participants might have been able to recall A–B items but withheld them due to uncertainty about their membership in the first-list, this additional test cue should have greatly reduced any such under-reporting. The fact that forgetting still occurred indicates that the decreased recall likely reflects genuine retrieval failure for A–B items. A cue-dependent retrieval failure of this sort is consistent with the potential for non-inhibitory blocking processes to contribute to forgetting measured on same-probe tests (for a discussion, see Schilling, Storm, & Anderson, 2014). It is also consistent with the numerically larger forgetting effects observed on the same- and independent-probe tests in the retrieval practice conditions (M = 17% vs. 12% on the same- and independent-probe tests, respectively, in Experiment 1; M = 14% vs. 6% in Experiment 2). Thus, whereas the independent-probe test likely reflects inhibition of the target itself, the same-probe test may be produced by both inhibitory and non-inhibitory factors, as has been suggested previously (Anderson et al., 1994; Anderson & Levy, 2007; Schilling et al., 2014). These findings also echo reported dissociations within the retrieval suppression literature suggesting that active inhibition (via direct suppression) reliably generates cue-independent forgetting, whereas requiring participants to generate or learn novel, interfering associations with the original cues as a form of distraction does not (Bergström, de Fockert, & Richardson-Klavehn, 2009; Wang, Cao, Zhu, Cai, & Wu, 2015).

In the current retroactive interference design, participants encoded two responses associated with each cue, ensuring that all pairs conformed to the A–B, A–C structure typically associated with interference. Our main manipulation, which enabled us to detect retroactive interference, involved a parametric manipulation of the number of anticipation trials on A–C associations (0, 1, 6, or 12 repetitions; see, e.g., Barnes & Underwood, 1959). This design isolated the impact that these interpolated anticipation trials (and, in Experiment 2, the retrieval and feedback components of anticipation) had on retroactive interference, while holding representational structure constant (i.e., a single cue with two associates). It is noteworthy, however, that many conventional retroactive interference designs often included a condition in which A–B learning is followed either by rest or by unrelated pairs (e.g., D–E pairs) that should produce no specific interference. In such a design, retroactive interference is measured by comparing a condition in which the cue had a single associate to one in which the cue had two associates (see, e.g., Anderson & Neely, 1996, for a discussion). As such, it would then reflect a mixture of the impact of changing the associative structure (i.e., a cue with one vs. two learned responses) and the impact of additional interpolated anticipation attempts on the A–C association. Such a comparison would likely produce an even larger effect than the one we presently observed. It is theoretically possible that encoding A–B pairs with the anticipation method might have insulated participants against retroactive interference that would arise by simply encoding a new association to a cue (as would have been detected by the difference between A–B alone and encoding A–B and A–C, without any anticipation attempts on the latter). This possibility cannot be excluded by our data. However, because our theoretical focus was on whether an initial retrieval insulates items against retrieval-related inhibitory processes – and because this comparison would not involve interpolated retrieval – the omission of this additional control is not relevant to our key hypotheses.

Taken together, these findings indicate that inhibitory control processes believed to contribute to retrieval-induced forgetting also contribute to measures of retroactive interference collected in the A–B, A–C interference paradigms that utilise the method of anticipation. Our findings also reveal the contribution of two seemingly distinct mechanisms to retroactive interference: one that is cue independent (inhibition) and one that is cue dependent, consistent with associative blocking (McGeoch, 1942). If this interpretation is correct, these findings suggest that an initial retrieval of recently encoded material does not fully insulate the retrieved content from inhibitory processes at work in retrieval-induced forgetting. Indeed, an initial retrieval also did not insulate items from cue-dependent interference processes either. Next, we consider these findings in relation to the literatures concerning the protective effects of retrieval, as well as forgetting phenomena more broadly.

### Relation to other findings

The present findings differ from those reported by Kliegl and Bäuml (2016) who found that an initial retrieval of a set of items protected them from retrieval-induced forgetting. In contrast, we found robust retroactive interference that is likely to have a significant retrieval-induced forgetting component. These findings suggest that there are important limits on the generality of Kliegl and Bäuml’s conclusions about the insulating effects of retrieval. They also raise the question of what might account for the divergent findings.

We suggest that one critical feature of Kliegl and Bäuml’s (2016) design setting the stage for the abolition of retrieval-induced forgetting might have been their use of free recall to prompt the initial round of retrieval. For example, in their experiment focusing on retrieval-induced forgetting, they had participants study categorised word lists and then do category-cued free recall (what they call the non-selective retrieval phase) for each of the categories prior to the selective retrieval phase. Another group simply restudied the category members instead of receiving the initial free-recall task. Whereas the latter group showed retrieval-induced forgetting, the former did not. Importantly, however, Zaromb and Roediger (2010) demonstrated that free recall of lists facilitates the organisation of the list more so than that which is effected by a simple restudy attempt. In Kliegl and Bäuml’s study, this could have prompted the development of inter-item associations between exemplars of a category and increased the development of a structured retrieval plan.

Given that the formation of such integrated associations has been shown to eliminate retrieval-induced forgetting (Anderson & McCulloch, 1999; Goodmon & Anderson, 2011), their development seems to be a plausible mechanism by which the initial category cued free recall eliminated the forgetting effect. In contrast, because the method of anticipation conducted on first-list A–B pairs in the current study focused on individual responses to individual stimuli, the opportunity for the formation of such associations was greatly constrained, thereby eliminating integration as a benefit of retrieval. If, however, retrieval protects memories by triggering the storage of distinctive contextual features, as Kliegl and Bäuml (2016) argued, the method of anticipation should have eliminated retrieval-induced forgetting. Indeed, Kliegl and Bäuml’s emphasis on reducing within-list interference by reducing competition through distinctive encoding emphasises retrieval’s effects on individual memories, not what links them together – a factor which was not necessarily maintained throughout their procedure. The current findings, therefore, suggest that the organisational benefits of retrieval and not the storage of distinctive temporal context could plausibly have been behind Kliegl and Bäuml’s findings. A similar interpretation also could be applied to their second experiment eliminating output interference, which used a similar free recall procedure.

Although list organisation may account for Kliegl and Bäuml’s (2016) findings, it may not factor into similar results reported by Racsmány and Keresztes (2015). In Racsmány and Keresztes’s study, participants encoded six examples of each of eight taxonomic categories. They were then given the opportunity to retrieve all of the studied items with a category-plus-stem cued-recall test (e.g., FRUIT-A__) prior to performing selective retrieval practice (e.g., FRUIT-AP__) as is done in the standard retrieval-practice procedure. A final test probed participants’ memory yet again with category-plus-stem cues (e.g., FRUIT-A__). They found that, whereas selective retrieval practice facilitated the later recall of practiced items, it did not harm recall of unpracticed members of the practiced categories. Thus, the initial opportunity to retrieve all the studied items prior to selective retrieval practice abolished retrieval-induced forgetting, and this arose even though that initial recall did not involve a category-cued free recall test, as was used by Kliegl and Bäuml (2016). Although category-plus-stem cued-recall testing on the initial test theoretically could induce retrieval organisation effects of the sort established by Zaromb and Roediger (2010), this has not been addressed empirically; such organisation effects seem, however, less plausible, given that testing focuses participants’ attention on specific target exemplars with letter stems.

The differing outcomes may, instead, owe to another potentially important difference between our experiments and those of Racsmány and Keresztes (2015). Participants in Racsmány and Keresztes’s experiments performed an initial retrieval test on *all* of the studied items, not just on the competitors. As a result, the potential insulating benefits of an initial retrieval attempt on competitors were confounded with its impact on the future retrieval-practice targets (this also applies to Kliegl & Bäuml, 2016). In contrast, the present study only gave an initial retrieval test on the competing items (A–B pairs) prior to the critical retrieval practice trials thought to induce inhibition (i.e., on A–C items). It is possible that in Racsmány and Keresztes’s study, the selective retrieval-practice trials were rendered trivial, due to the heightened accessibility of to-be-practiced items, owing to their recent retrieval. Thus, the initial retrieval may have reduced any need to resolve competition via inhibition. By this hypothesis, retrieval need not do anything to insulate competitors from inhibition, and their findings might instead reflect the competition dependency of inhibitory control (Anderson, 2003). Perhaps compounding matters further, Racsmány and Keresztes’s (2015) initial test cued with letter stems, potentially leading participants to place greater weight on the letter cues during later retrieval practice (e.g., AP__), reducing competition from other category exemplars (for a discussion, see Anderson, 2003). The current findings make it clear that when only competitors are retrieved – and are retrieved in a way that does not reduce the competitiveness of the later retrieval practice process – robust retrieval-induced forgetting is observed.

Nevertheless, the current data may be consistent with a role of retrieval in insulating items from inhibition, under certain assumptions. Perhaps retrieval of A–B items during anticipation trials did insulate the retrieved items from inhibition, but this protection was partial or incomplete. For example, if we had included another condition in which participants were trained on A–B pairs and then received extra study exposures instead of retrieval-based anticipation trials, we might have found larger retroactive interference effects than we did with the anticipation procedure. This pattern would suggest that while retrieval does not fully insulate items against either interference or inhibition, it may nevertheless produce uniquely protective effects. Such an account would still be compatible with Kliegl and Bäuml’s (2016) contextual distinctiveness hypothesis, with additional assumptions to explain why, in the current study, items remained vulnerable to interference and inhibition, unlike what was found in their study. This alternative hypothesis should be tested in future work. Nevertheless, the current findings indicate that the strong view holding that a single retrieval attempt of a competitor fully insulates items from inhibitory processes underlying retrieval-induced forgetting clearly cannot be maintained.

Our finding of retroactive interference on the same-probe test after extra study repetitions on A–C items extends similar findings reported by Halamish and Bjork (2011). These authors had participants encode A–B pairs either by three study repetitions or by a single study and two retrieval practice attempts. This was then followed by a single encoding of either A–C (interference) or D–E (control) items, and a final test on A–B pairs. Halamish and Bjork reported reliable retroactive interference on A–B pairs (calculated as control – interference recall) that was larger when they had been studied (M = 33%) compared to when they had been tested (M = 20%). Thus, A–B pairs always suffered retroactive interference in their hands, but they did so less when they had been retrieved initially. Importantly, however, retroactive interference remained significant even after an initial retrieval test.

Our finding that extra study induced retroactive interference echoes that observation, while extending it in three ways. First, because Halamish and Bjork (2011) tested A–B items (e.g., KNEE-BEND) with a cue that matched both the A–B pair (KNEE-B_N_ for BEND) and the second-list A–C pair (KNEE-B_N_ for BONE), they cannot distinguish true retrieval failure of A–B items from failed list-discrimination for otherwise highly accessible pairs. In contrast, we provided a distinctive letter stem on our same-probe test in Experiment 2, so our retroactive interference effect cannot be explained by failed list discrimination and likely reflects a true accessibility deficit. Second, whereas Halamish and Bjork compared the presence of interference versus its absence (A–B, A–C vs. A–B, D–E), our procedure always included A–C interference learning, while parametrically manipulating the degree and form of A–C training. Finally, we demonstrated that the retroactive interference effect following extra A–C study exposures did not generalise to an independent probe test. Together, our findings reinforce the existence of a cue-specific form of interference leading to a retrieval deficit that survives an initial retrieval of A–B items. This conclusion is consistent with the observation that the overwhelming majority of research on retroactive interference from the classical interference era used the method of anticipation to train A–B pairs yet observed robust retroactive inference that increased with the degree of interpolation (see, e.g., Anderson & Neely, 1996; Barnes & Underwood, 1959; Postman, 1971). Thus, retrieval is unlikely to fully insulate against retrieval-related inhibitory processes underlying retrieval-induced forgetting or against cue-specific interference mechanisms.

Other published findings similarly suggest that an initial retrieval attempt does not insulate against inhibitory control, including work on retrieval suppression. As in the current experiment, studies of retrieval suppression require that participants first acquire a set of paired associates (e.g., words or pictures) using the method of anticipation. This is then followed by a task that presents participants reminders of studied pairs and asks them to retrieve the associated item (i.e., Think trials) or to actively suppress retrieval of the associated item (i.e., No-Think items). On a final memory test after this Think/No-Think phase, participants generally recall No-Think items more poorly relative to both Think items and also baseline items that had been similarly well learned but had neither been suppressed nor retrieved in the interim (see Anderson & Hanslmayr, 2014; Levy & Anderson, 2002, for reviews). When achieved through active inhibition, the below-baseline suppression-induced forgetting effect typically generalises to independent test probes (Anderson & Green, 2001) and also is supported by an inhibitory control process that suppresses mnemonic representations mediated by the hippocampus (Anderson et al., 2004; Anderson & Hanslmayr, 2014; Anderson, Bunce, & Barbas, 2016; Hulbert, Henson, & Anderson, 2016). That significant forgetting is observed despite repeated retrieval of the initial pairs indicates that retrieval does not fully insulate against inhibitory effects on memory (see also Wang et al., 2015).

Although we have argued that an initial retrieval attempt does not necessarily fully insulate against retroactive interference or retrieval-related inhibitory processes, our specific intention was to address the issue of whether retrieving individual items renders them resilient, perhaps either by facilitating their consolidation (Antony et al., 2017) or by storing contextually distinctive features with the item (Kliegl & Bäuml, 2016). As such, our conclusions should be restricted to those circumstances, which makes them compatible with demonstrations that retrieving sets of material renders those sets resilient, either by changing their organisation/integration (Zaromb & Roediger, 2010) or by a related change in list segregation (Abel & Bäuml, 2014; Bäuml & Kliegl, 2013; Halamish & Bjork, 2011; Szpunar et al., 2008). It seems possible that the effort to freely recall a large set of items induces changes in organisation that eliminate inhibition and increase the ability to discriminate one list from another. What seems less probable, based on the current findings, is that this resistance to interference reflects intrinsic changes to individual items that increases their durability, though a partial protective influence cannot be ruled out at present.

## Concluding comment

Retrieval plays a fundamental role in shaping the fate of experience in memory, both dramatically enhancing the retention of retrieved content and disrupting retention of traces that evoke competition during retrieval (e.g., Bekinschtein et al., 2018). Recently, there has been increasing interest in how the benefits and costs of retrieval might interact with one another, leading to the important question: Does retrieving a memory render it resilient to retrieval-based forgetting processes that might be applied to the retrieved memory on later occasions? If so, what is the mechanism behind this protective influence, and under what circumstances does it operate? Using a retroactive interference design with anticipation training, we explored the recent suggestion that retrieval insulates items from retrieval-induced forgetting by storing a distinctive representation of context that renders it non-interfering and less necessary to inhibit.

Our findings do not provide strong support for this theoretical proposal. Rather, even when competitor items were trained through retrieval practice (i.e., the method of anticipation), they nonetheless showed forgetting that was both cue independent and retrieval specific, pointing to an important role of retrieval-related inhibitory processes in the classical phenomenon of retroactive interference. Our data thus indicate that retrieved memories are not fully insulated from inhibition and argue that another explanation of protective benefits of retrieval is needed. We suggest that at least some demonstrations that retrieval-induced forgetting and output interference may be eliminated by an initial retrieval may reflect the tendency for free recall to trigger integration amongst list members – a factor already known to eliminate the effect (Anderson & McCulloch, 1999). Other findings suggestive of an insulating effective of retrieval instead may reflect a confounding of the effects of an initial retrieval attempt on the accessibility of practice targets and any insulating properties on competitors. If these hypotheses are correct, it suggests that while recall may sometimes protect against forgetting, it does so via mechanisms that do not, in themselves, require retrieval to occur (e.g., integration, priming of practiced targets). The mechanisms by which retrieval sometimes reduces retrieval-induced forgetting thus remain to be more fully understood.

## Notes

1 In Experiment 2, participants received a single letter word-stem on the SP test in order to better match the retrieval conditions between test types.

2 An analysis of intrusions of C responses on the SP test for B responses when cued by the shared stimulus revealed no significant differences between the retrieval practice levels.
